# Point-of-use sweat biosensor to track the endocrine–inflammation relationship for chronic disease monitoring

**DOI:** 10.2144/fsoa-2020-0097

**Published:** 2020-10-22

**Authors:** Sayali Upasham, Ashlesha Bhide, Kai-Chun Lin, Shalini Prasad

**Affiliations:** 1 Department of Bioengineering, University of Texas at Dallas, Richardson, TX 75080, USA

**Keywords:** biosensor, cortisol, electrochemical, endocrine, inflammation, sweat, tumor necrosis factor-α

## Abstract

**Aim:**

The hypothalamic–pituitary–adrenal axis is involved in maintaining homeostasis by engaging with the parasympathetic nervous system. During the process of disease affliction, this relationship is disturbed and there is an imbalance driven response observed.

**Materials & methods:**

By monitoring the two key components involved in these pathways, cortisol and TNF-α, the manifestations of chronic stress on the body’s homeostasis can be evaluated in a comprehensive manner. This work highlights the development of an electrochemical detection system for the two biomarkers through human sweat.

**Results:**

Limit of detection and dynamic ranges are 1 ng/ml, 1–200 ng/ml for cortisol and 1 pg/ml, 1–1000 pg/ml for TNF-α.

**Conclusion:**

This wearable system is designed to be a point of use, chronic disease self-monitoring and management platform.

## Lay abstract

When the body is under stress, a lot of physiological processes work toward bringing the body back to its normal state. Cortisol, a stress hormone and TNF-α, a protein related to inflammation, are direct products of these physiological processes. The goal of this work is demonstration of a biosensor that is capable of tracking these molecules in human sweat. The significance lies in simplifying disease diagnostics, in other words, to detect and monitor diseases. This system is designed to be a wearable that will track the levels of cortisol and TNF-α and use it as an indicator of the user's health status.

Understanding the relationship between the endocrinal pathway for stress regulation and the inflammation pathway is key to understanding the physiological effects and the extent of damage inflicted during periods of exposure to stress. The hypothalamic–pituitary–adrenal (HPA) axis is a prime component of the endocrinal system to prepare the body to react to a stressful episode. The response to stressful stimuli is nonvoluntary and is aimed at bringing the body back into homeostasis via a process known as allostasis. A detailed summary of the response to stress is illustrated in Figure 1. The signal originates in the hypothalamus, specifically in the hypothalamic paraventricular nucleus. The neurons in response to this produce corticotropin-releasing hormone. Corticotropin-releasing hormone production signals the pituitary gland to release adrenocorticotropic hormone into the blood stream. Once in the blood stream, as the name of the hormone suggests, it activates the receptors present in adrenal gland – specifically in the adrenal cortex. This houses cells that are capable of steroidogenesis and with the adrenocorticotropic hormone-mediated activation, start producing glucocorticoids, like cortisol. Cortisol is an anti-inflammatory corticosteroid, which is responsible for performing allostasis by activating various pathways responsible for a decreased inflammatory response in the target organs. One of the pathways functions by reducing the adaptive immunity effector cells, in other words, T cells, by apoptosis and the other pathway performs tightening of the tight junctions in endothelial cells to prevent transport of peripheral immune cells from entering the blood-brain barrier. However, the main response of cortisol mediated control of inflammation is through the glucocorticoid receptor (GR) controlled genomic immunosuppression, which reduces the production of pro-inflammatory cytokines like TNF-α [1,2]. There is a presence of a negative feedback mechanism that is controlled by the affinity of corticosteroids to GRs. After sufficient production, the receptor is saturated and signals the HPA axis components to stop production of GC activating hormones. With the production of cortisol as a response to stress, the inflammation pathway is stimulated as demonstrated in Figure 1 (right side). The glucocorticoid receptor has a major role in the processes responsible for cell homeostasis. There are various modes of action by which the GC receptors operate. In this research, we focus on capturing the pathway that is responsible for controlling the production of TNF-α. Normally, GR is present in the cytoplasm; once activated with corticosteroid, the bound proteins dissociate and it enters the nucleus. Figure 1 depicts a red cylinder, which is the HSP90 chaperone protein and the two circles are the other accessory proteins involved with activation and transport of the GR from the cytoplasm to the nucleus. The GR regulates genomic expression by dimerizing to glucocorticoid response elements in the target genes and affecting the transcription/translation of the mRNA to produce cytokines. Due to this effect, the production of pro-inflammatory cytokines like TNF-α, IL-6 and IL-1β are decreased and the production of anti-inflammatory cytokines such as IL-10 and TGFβ is increased [3,4]. The interconnection of these two pathways is of diagnostic importance to many disorders. An impairment of these regulation pathways is highly associated with mortality. In case of patients suffering from Crohn’s disease, findings suggest that the inflammation pathway is disturbed and there is an overexpression of TNF-α. Similarly, in the case of Irritable Bowel Disease there is an imbalance in the sympathetic control of the adrenal gland activity. The triggers for generating severe symptoms in both these conditions were observed to affect the HPA axis functioning first, thus resulting in lower cortisol production [5]. A report also suggests that the elevation of pro-inflammatory cytokines and corticosteroid levels has negative effects on the cognitive functioning and hippocampal structure of older adults [6]. All these examples highlight the interconnectivity of the two pathways and the need to monitor the direct products from a chronic disease diagnostics and monitoring aspect. The effective solution for offering a convenient platform for self-monitoring is using sweat for biomarker quantification. Human sweat based sensing platforms offer significant advantages over traditional and gold standard methods of detection. Physiologically, cortisol and TNF-α are expressed in sweat in the ranges of 8–141 ng/ml [7] and 9–362 pg/ml [8], respectively. There have been significant advances in the field of developing sweat-based cortisol sensors for investigation into the effects of stress on the body. TNF-α has been explored as a key to understanding the regulatory processes in the body upon the onset of inflammation. Table 1 describes the different biosensors developed for tracking TNF-α and cortisol in various biofluids, which are aimed toward eventually becoming wearable technology. TNF-α detection in sweat has not yet been extensively researched and is an upcoming area of interest among the researchers in the field of wearable diagnostics. However, in the past few years, researchers have looked at understanding the fluctuation of cortisol and TNF-α levels in blood/serum toward characterization of phenotypes of multiple diseases [9,10]. The levels of cortisol have been known to correlate with the sweat levels with levels being independent of sweating rate [11]. Similarly for TNF-α, it has been reported that sweat biomarker levels closely mirror the blood/serum levels and can be easily detected using wearable patches [8]. By tuning the sensor to capture changes in the biomarker levels withing the physiologically relevant levels of cortisol and TNF-α, this platform offers a novel approach toward understanding the effect of circadian dysregulation on the inflammatory response of the body. This would also enable early detection of circadian dysregulation and understanding its connection with the etiology and pathophysiology of disorders.

**Figure 1.**
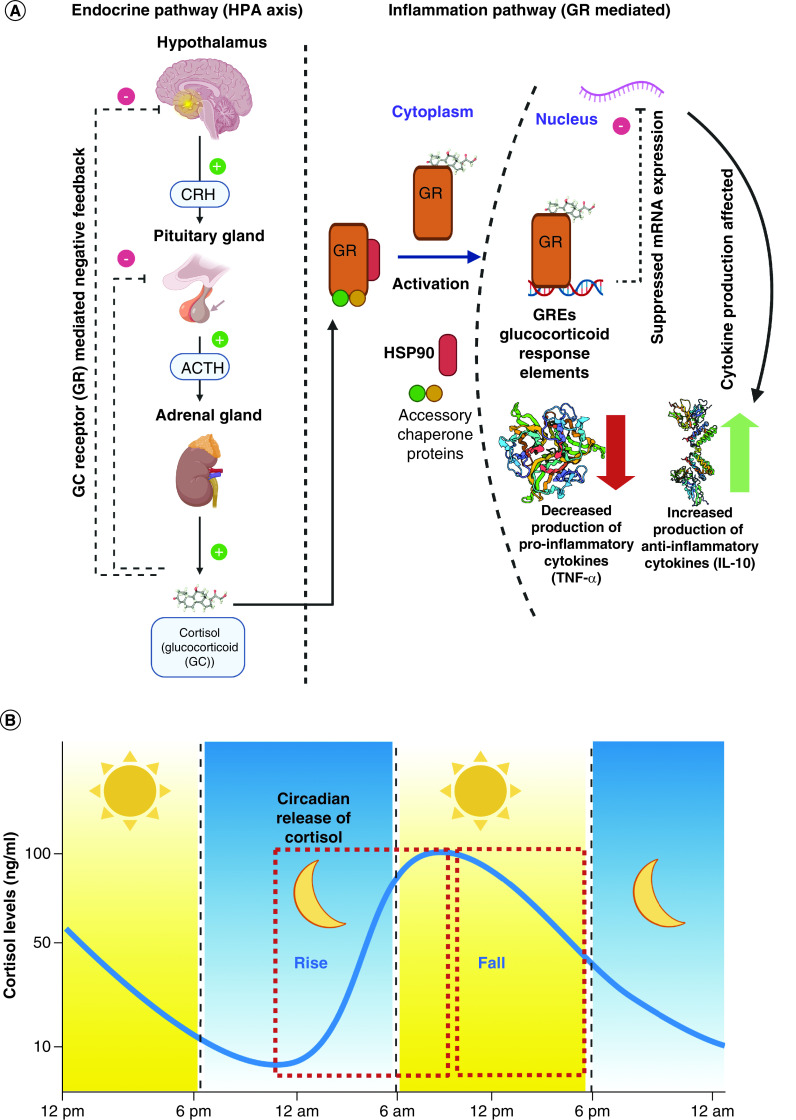
Pathways for circadian-inflammatory relationship. **(A)** Depicts the relationship between the endocrinal HPA axis pathway and inflammation pathway (TNF-α), **(B)** Schematic depicting diurnal cycling of cortisol over the period of 24 h. HPA: Hypothalamic–pituitary–adrenal.

**Table 1.** Biosensor technologies for detection of TNF-α and cortisol.

Sensor description	Biomarker and biofluid	Type of detection	LOD	Ref.
Polypyrrole based gold screen printed electrode (enzyme labelled)	TNF-α, bovine calf serum	Potentiometric	10 pg/ml, compared with commercial ELISA	[12]
Gold interdigitated electrode capacitor arrays	TNF-α, PBS	Label-free capacitance measurements	32 pg/ml	[13]
Affinity based gold electrode functionalized using CMA	TNF-α, artificial saliva, human saliva	Chronoamperometry	1 pg/ml	[14]
Stretchable and disposable 3D micropatterned elastomer-based electrode	TNF-α, human serum	Electrochemical (potentiometric and impedimetric)	100 fM	[15]
Label-free chronobiology tracking system	Cortisol, sweat	Impedimetric	1 ng/ml	[16]
MoS_2_ nanosheet based flexible electrode	Cortisol, sweat	Impedimetric	1 ng/ml	[17]

CMA: Carboxymethylaniline; ELISA: Enzyme-linked immunosorbent assay; LOD: Limit of detection; MoS_2_: Molybdenum disulphide; PBS: Phosphate-buffered saline.

Multiplexed biomarker detection has gained considerable visibility in point-of-use testing arena in the recent years, owing to its capability in enhancing the diagnostic detection process of an underlying condition with precision and efficiency through rapid on-site analysis of bodily fluids [18]. Multibiomarker detection platforms provide an in-depth clinical understanding of the processes occurring within the body with a certainty that a condition is indeed present in an individual; single biomarker analysis may not prove to be effective in providing accurate diagnosis [19,20]. Multiplexed point-of-care testing, through its capabilities, has paved the way for home-based, personalized patient-centric systems for the management of chronic and acute diseases in resource limited settings [21]. Conventional testing methods are tedious, constrained to laboratory and hospital setups, require trained personnel and require prolonged analysis times [22]. Wearable technology is expected to show a global growth rate of 38% in the next decade and will continue to evolve with the advent of smart-watches, patches, clothing and fitness trackers [23]. Real-time, continuous health monitoring is one important feature that is being explored in the research space of multiplexed biomarker detection to provide an account of an individual's health status for the healthcare provider to administer accurate treatment and drug therapy. Herein, we describe a proof-of-concept multiplexed detection of stress biomarker cortisol and inflammatory cytokine TNF-α on flexible, body conforming substrate toward integration on a wearable platform. The choice of substrate is a key parameter to be considered while designing flexible biosensing platforms. The desired features of the selected substrate are bendability, foldability, stretch ability, portability, skin-conformability, disposability and being lightweight [24]. We have utilized a nanoporous polyamide membrane that allows for sweat wicking through its intercalations, sensitive detection due to enhanced charge storage through its nanoconfinement properties and selective detection of target biomolecules from the biomolecular sieving properties [25].

In this work, we have demonstrated the functionality of biosensing platform in detecting dual biomarkers – cortisol and TNF-α in human sweat toward the development of a multiplexed point of use device. Additionally, the long-term temporal stability of the cortisol biosensor in detecting the simulated rise and fall in cortisol levels through the 6-h sleep cycle has been demonstrated. Furthermore, COMSOL Multiphysics® simulation highlight the electrode design features and fluid wicking pattern of membrane for design optimization of the sweat based platform. The cross-reactivity performance demonstrates that the sensor is highly specific for the target biomarker. Thus, this novel platform shows the feasibility of tracking the endocrine–inflammation relationship toward chronic disease diagnostics using sweat.

## Materials & methods

### Reagents & materials

Cortisol antibodies and cortisol molecules were procured from Abcam (MA, USA). The TNF-α antibody and antigen, thiol linker used dithiobis(succinimidyl propionate) (DSP) and dimethyl sufloxide were purchased from Thermo Fisher Scientific Inc. (MA, USA). Milipore de-ionized water (conductivity – 18 MΩ cm) was used to prepare the solutions. Nanoporous polyamide membranes were obtained from GE Healthcare Life Sciences (NJ, USA). Pooled human sweat was procured from Lee Biosolutions (MO, USA). No animal or human subjects were tested in this work.

### Sensor fabrication

The gold sensors were fabricated in-house utilizing the facilities provided by the cleanroom at University of Texas at Dallas (TX, USA). Cryo e-beam evaporator was used to deposit a thin gold film (150 nm) on the nanoporous polyamide membrane. Shadow masks were placed on the electrode surface during deposition to create the interdigitated gold electrode pattern on the sensors. This was then used for functionalization followed by testing. The sensor is designed to operate with ultra-low volumes of sweat to enable passive sample collection. The volume of sample needed is 3 μl, which aligns with the passive eccrine sweat rate and sweat production amount at rest [26].

### COMSOL Multiphysics software simulations

Finite element analysis was carried out utilizing licensed software version of COMSOL Multiphysics v5.4. The module used for performing simulations are transport of diluted species in porous media, electrostatics and primary current distribution. 3D multislice plots for electrolyte potential, 1D plot group for current density and wicking simulations were exported through the software.

### Fourier-transform infrared spectroscopy experimental details

Infrared spectra of functionalized electrode were recorded with a Thermo Scientific Nicole iS50 FT-IR using an Attenuated Total Reflectance (ATR) stage. The tool was equipped with a deuterated triglycine sulfate detector and KBr window. A Harrick VariGATR sampling stage with a 65° Germanium ATR crystal was used in this study. ATR-Fourier-transform infrared spectroscopy specimens were prepared by deposition solutions on polyamide membrane as substrate. The contact area was about 1 cm^2^. All spectra were recorded between 4000 and 700 cm^-1^ with a resolution of 0.5 cm^-1^ and 256 scans.

### Experimental detail for electrochemical analysis

Fabricated gold electrodes were tested for baseline stability and then employed for functionalization. This was performed by incubation with DSP/dimethyl sufloxide for 1.5 h followed by incubation of 10 μg/ml of antibody solution for 30 min. Incubation times were optimized using wicking simulation and baseline studies. Functionalized electrodes were utilized for testing by building an immunoassay using increments of target dose concentrations. During measurements of electrochemical impedance spectroscopy (EIS), a 10 mV AC potential bias was applied to the working electrode against the reference electrode and the response was recorded. These electrochemical measurements were carried out using Gamry Reference 600 potentiostat (Gamry Instruments, PA, USA). Dose concentrations of target biomarkers were tested within the physiologically relevant concentrations. These dose concentrations were prepared by artificially spiking the human sweat and analyzing the data as change from the baseline. Human sweat already has proteins, steroids and other molecules present in the solution. The process in which the biofluid was spiked was by adding a known concentration of target biomarker ranging from 1–200 ng/ml for cortisol and 1–1000 pg/ml for TNF-α. These spiked biofluid doses were then introduced on the functionalized sensor surface and the change in response from the blank human sweat was recorded as sensor response. The optimal frequency was 100 Hz, where maximum capacitive behavior was observed.

### Experimental detail for long-term studies

For the long-term studies, single frequency EIS was performed over the study time period (60 min and 6 h) and the sensor was loaded at 5-min intervals. The frequency of operation was set at 100 Hz. For the short study of 60-min duration, increments of cortisol dose concentrations ranging from 1–100 ng/ml were loaded on the sensor surface. To capture the rise, concentrations of 1 and 100 ng/ml were loaded consecutively on the sensor surface. And to capture the fall, concentrations of 100 and 10 ng/ml were loaded on the surface consecutively. The cumulative impedance response was then recorded using single frequency EIS. Similarly, for the long term 6-h continuous study, concentrations were ramped up from 20 to 100 ng/ml in increments of 20 ng and fall was captured by loading concentrations of 80 ng/ml followed by 60 ng/ml.

### Statistical analysis

Data is represented as mean ± SEM with an n = 3, where n is the number of biosensor replicates tested. The interassay and intra-assay variations are less than 10% which is compliant with Clinical and Laboratory Standards Institute guidelines [27]. Statistical analysis for dose dependent response was performed using analysis of variance (ANOVA) followed by Tukey’s test to establish significance. Unpaired *t*-test was employed when the test for significance was between two groups. The confidence interval was fixed at 95% and α was 0.05. The analyses were performed using GraphPad Prism version 8.01 (Graph Pad Software Inc., CA, USA).

## Results & discussions

### Sensor substrate characterization

**Figure 2.**
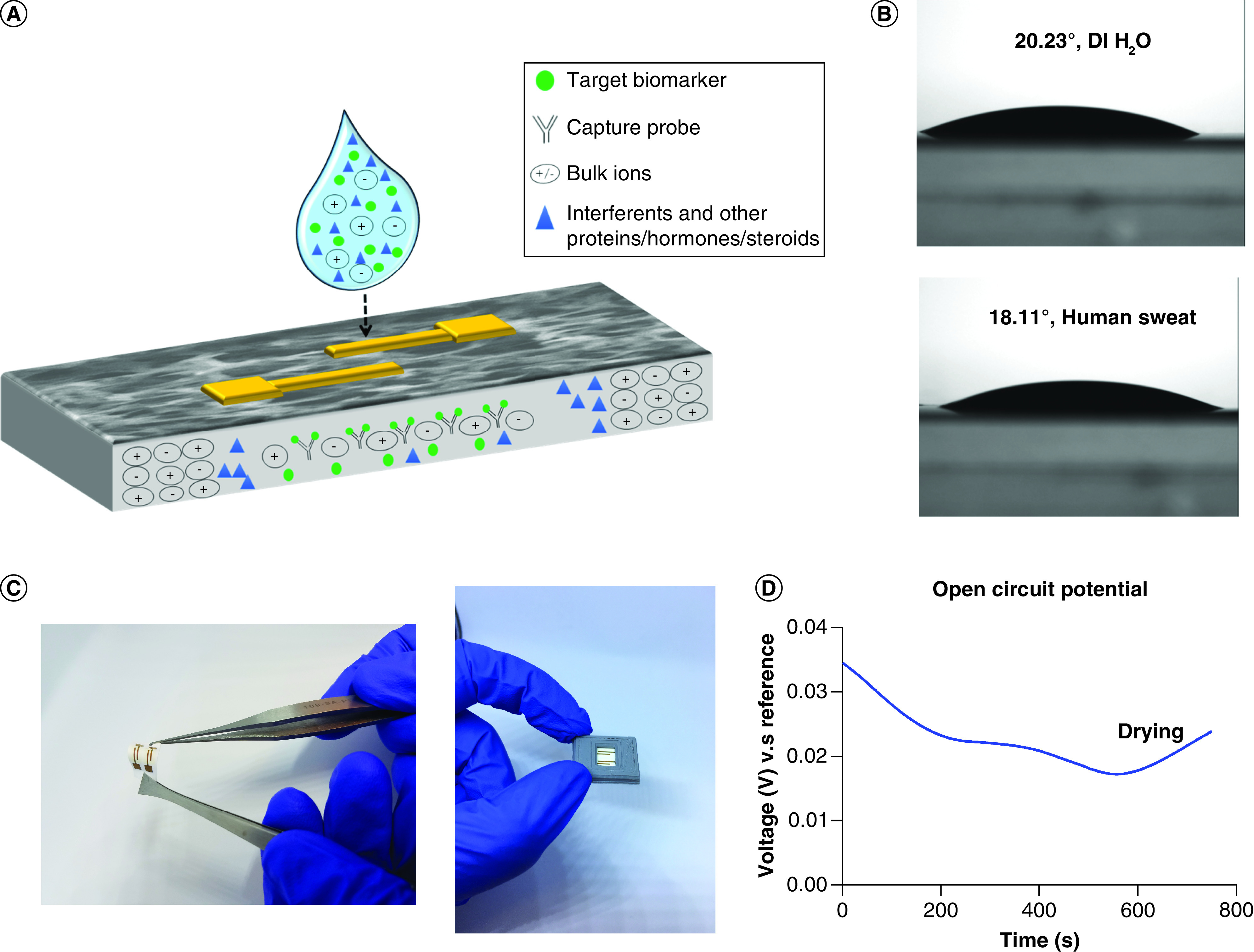
Sensor characterization. **(A)** Schematic depicting the wicking of sweat with target biomarkers (cortisol and TNF-α) on the nanoporous membrane, highlighting selective molecular confinement, **(B)** Contact angle studies using DI water (top) and human sweat (bottom) on membrane, **(C)** Sensor pictures to highlight flexibility of platform and sensor prototype with case and **(D)** Open circuit potential for electrochemical stability. DI: De-ionized.

The focus of this work is to demonstrate the functionality of a novel, flexible sweat based sensing platform for quantifying the concentrations of biomarkers, cortisol and TNF-α. The substrate employed to make this platform is a nanoporous polyamide membrane. A two-electrode interdigitated system is chosen as the electrode design and fabricated using thin film gold deposition. The physical properties of the nanoporous membrane are highlighted in Supplementary Table 1. Nanoporous platforms offer significant advantages over porous materials. They increase the overall surface area of interaction between the target molecule and receptor, in other words, capture probe. This work is based on an affinity sensing mechanism by using electrochemical detection modality. The gold electrode surface is treated to immobilize the detection probe, in other words, the antibody, using a thiol linker chemistry. The analyte present in sweat is introduced on the sensor surface and it wicks through the membrane to interact with the bioactive components and generate a signal response. The advantage of having a nanoporous membrane is that it provides selective molecular confinement based on size and diffusion kinetics [28]. A schematic of this phenomenon is presented in Figure 2A. The schematic describes the process of detection of target biomarker in a complex biofluid sample matrix, which in this case, is human sweat. Sweat is a complex mixture of proteins, steroids, hormones, electrolytes and other interferent molecules. The blue droplet of sweat is loaded on to the functionalized sensor surface, with the antibodies concentrated on the gold surface of the sensor. The sensor surface depicted as a grey matrix in the figure is a magnified cross-section of the sensing membrane. With the nanopore based filtration, there is enhancement in the selectivity of the sensor response. Also, this reduces the overall noise of the system and improves the selectivity and sensitivity. The principle of wicking in this nanoporous membrane is capillary imbibition. Two main factors contribute to it, one is the transport of biomolecules normally a function of permeability and retention factor of the membrane and second is biomolecular confinement, which is associated with the pore packing structure of the membrane. The retention factor describes the affinity of the dye to the solid phase, which is the nanoporous polyamide membrane. Zone separation of a liquid solute during wicking through the nanoporous membrane highlights the ability of the membrane to filter the different components in the liquid phase. The red dye solution has approximately four components, thus the formation of three or more separation zones indicates that the chosen substrate is able to perform density dependent separation of the mixture. The formation of zones of separation highlighted in Supplementary Figure 1 and shows the density-dependent filtration performed in the membrane. The biomolecular transport is a function of pore size, thickness, contact angle and pore diameter, whereas the confinement is driven by pore packing arrangement and density. Both of these factors are important measures while choosing a substrate to ensure maximum sweat wicking occurs during sample collection [16]. Permeability of the membrane drives the fluid transport across the membrane and influences biomolecule interaction. The goal is to leverage maximum lateral transport toward enhancing of biosensing outcomes of a detection platform, namely, limit of detection (LOD), sensitivity and range of operation [29]. The permeability calculated for the polyamide membrane was approximately 0.04 cm^2^ per second and the fluid coverage was identified to be 0.16 mm^2^ per second. The wicking profiles have been generated using COMSOL Multiphysics simulations, which highlights the rapid lateral transport of polyamide membrane. These profiles have been used to further calculate the optimal rate and time for fluid loading. From these calculations, the optimal time for complete sensor area coverage will be 16 s. Rapid wicking facilitates fast sample collection especially using a passive sweat collection method. Herein, no external stimulation is required by the user to generate a sample. Eccrine sweat volumes between 3 and 5 nl per gland per minute are produced passively by the sweat glands. Thus by employing this hydrophilic, rapid wicking membrane, this system can perform detection using ultralow volumes, in other words, 3 μl of human sweat [26]. All the conditions mentioned above are applicable to sweat based detection systems only if the surface is hydrophilic. This is because sweat is aqueous in nature. Contact angle studies illustrated in Figure 2B show the contact angles of de-ionized water and human sweat on the surface of the polyamide membrane. The contact angle was measured to be 20.23° for de-ionized water and 18.11° for human sweat, as shown in the figure. These studies were carried out at room temperature. Evaluation of contact angle, in other words, the angle measured between the tangents of a liquid–solid interface indicates the degree of wetting of the substrate [30]. A contact angle less than 90° indicates that the surface is hydrophilic. The acute angle results in this case confirm the hydrophilicity of the membrane, which also corresponds to high wettability. This is advantageous for developing wearable platforms as it facilitates rapid wicking of sweat throughout the membrane. Therefore, by employing this nanoporous membrane in the platform development, the dynamic range is extended due to optimal filtration, better signal resolution is promoted, bulk solution effects are reduced and higher sensitivity is observed. A picture of the sensing system, highlighting the flexibility aspect has been presented in Figure 2C.

#### Sensor stability

The electrochemical stability of the sensor is determined using open circuit potential. This is used to evaluate the inherent baseline potential and the potential fluctuations in the bare, nonfunctionalized electrode system [31]. The inherent potential can then be used to remove the offset created by the bare system during active measurement and determine the true binding responses from a normalized system. The potential of this system is averaged at 0.027 V or 2.7 mV, which is stable and has very low susceptibility for corrosion. The sensor system does not show any sharp peaks or sudden rise and fall in the system. The study is performed by drop-casting phosphate buffered saline solution on the sensor surface. Following 600 seconds, the membrane starts drying, which is characterized by the increase in the potential seen in the graph. Overall, the system indicates that it is electrochemically stable, not prone to corrosion and will not drive the electrochemical response.

### Sensor design & substrate simulations using COMSOL Multiphysics

**Figure 3.**
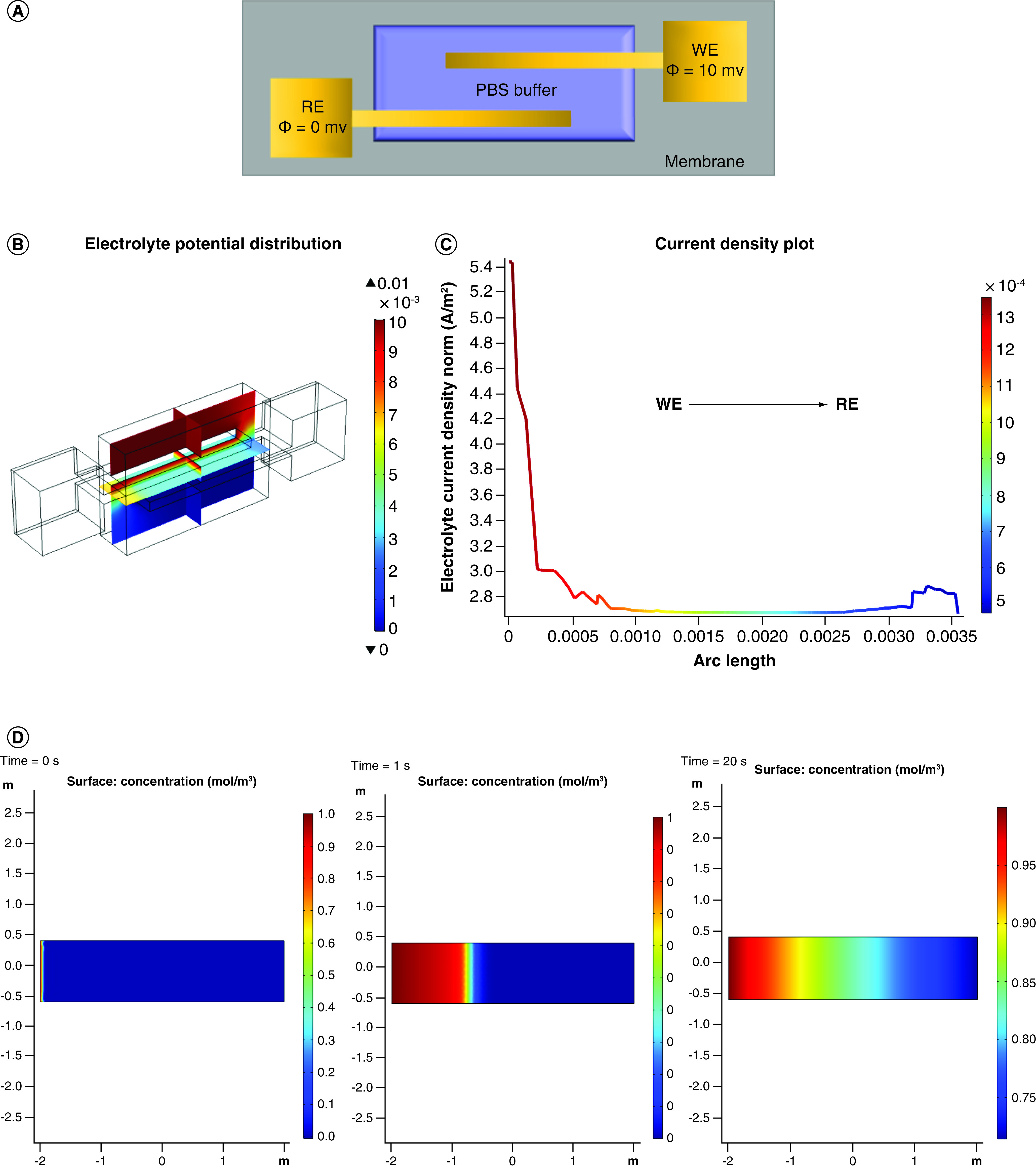
COMSOL Multiphysics^®^ simulations. **(A)** Electrode schematic depicting conditions for COMSOL simulations. **(B)** Multislice 3D graph depicting electrolyte potential distribution. **(C)** 1D line plot illustrating the current density distribution from WE to RE. **(D)** Simulation for wicking of solute over porous membrane at 0, 1 and 20 s. RE: Reference electrode; WE: Working electrode.

Finite Element Analysis (FEA) helps in visualizing the distribution of simulated electrochemical conditions. It can also be used to characterize the wicking pattern of the nanoporous substrate used in this work. Figure 3 demonstrates the various FEA results that were performed on the sensing system. Figure 3A highlights the geometry of the interdigitated electrode along with the appropriate boundary conditions. The sensor has two electrodes (reference electrode and working electrode) interdigitated geometry created by thin film deposition of gold. The working area is simulated by using a layer of phosphate saline buffer as an electrolyte for maintaining controlled conditions. The equations governing the simulations have been described in the Supplementary section 1. A 10 mV bias is applied to the working electrode and the distribution of electrolyte potential and current density was simulated. This is illustrated in Figure 3B & C. The distribution of electrolyte potential has been plotted as a multislice graph showing the cross-section view of the electrolyte. The maximum potential is concentrated around the working area with the gradient being created as we move from working to reference electrode area. The interdigitated electrode design is known to increase the overall capacitance of the system due to the increased surface area. Also, it provides enhanced sensitivity, lower detection limits, ability to operate with lower sample volumes and ease of fabrication [32]. The current density was extracted and plotted as a line plot extending from working to reference electrode. There is a sharp drop in the current density from 5.4 to 1.0 Am^−2^, highlighted by the current gradient present in Figure 3C. There are minimal parasitic current peaks indicative of an electrochemically stable sensing system.

Substrate optimization is of prime importance while designing optimal sweat based detection platforms. The substrate used for this study is a hydrophilic polyamide membrane. The membrane is designed to offer rapid wicking of sweat which enables instant sample collection and optimal interaction time between analyte and detection probes in the sensor system. Supplementary Table 1 summarizes the properties of this membrane. The surface capacitance has been presented as Supplementary Figure 2. The uniform distribution and value of inherent capacitance (values ranging in the Pico farad range) contributed by the membrane indicates that the membrane will not drive the capacitive behavior of binding. This is ideal, as the response is mapped as capacitive modulations and will be a direct function of binding between the target and detection probe. Another component related to simulations presented is performing FEA analysis to determine the wicking pattern and speed according to the given porosity of the membrane. This helps in optimizing the sample loading time and volume. Figure 3D highlights the lateral flow wicking profile of the polyamide membrane strip generated using COMSOL Multiphysics. It shows the progression of transfer of a dilute medium going from 0 to 1 molm^-3^ in a period of 20 s. This capillary wicking has been simulated using principles of Darcy’s law and diffusion kinetics [33]. The equations regarding the transport have been added to the Supplementary section 1. The assumptions related to the flow in the membrane strip model are also listed in the Supplementary data. The flow is modeled to be a bulk flow from one end of the strip to the other. It can be observed that rapid wicking occurs within the first 5 s and then once the membrane nanopores start saturating by filling up, the transport slows down. Within 20 s, it can be observed that there is transport of solute halfway through the membrane strip. The driving forces are capillary flow due to the pressure gradient created by the volume of liquid in the filled spaces versus the empty spaces [34]. This is modelled using the Lucas Washburn equation [35], which describes the capillary wicking in a channel inside a nanoporous membrane. Due to these fluid transport properties, the sensing system can successfully perform detection using ultralow sample volumes such as 3 μl. This is conducive to passive sampling for detection instead of active stimulation of sweat. Moreover, some of the other advantages of using this nanoporous membrane is that it reduces the biofouling that occurs at the electrode surface. Also being biocompatible, it nests with the user’s epidermis and does not create any local irritation at placement site.

### Binding chemistry characterization

**Figure 4.**
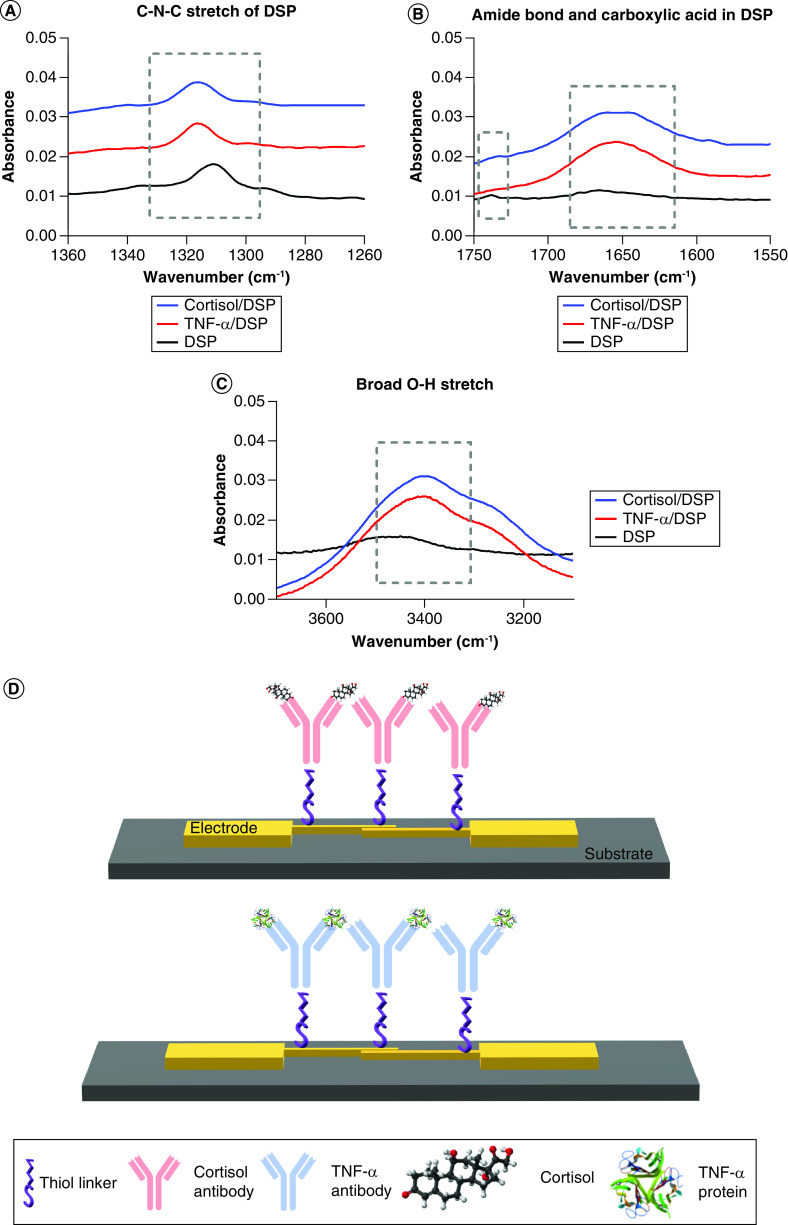
Binding chemistry characterization. Fourier-transform infrared spectroscopy spectrums validating the binding chemistry, **(A)** C-N-C stretch for DSP immobilization, **(B)** Amide bond and carboxylic acid highlight for protein immobilization, **(C)** Broad O-H stretch for antibody immobilization and **(D)** schematic depicting the immunoassay employed for performing affinity based detection for Cortisol and TNF-α.

ATR-IR analysis was performed to characterize the binding interaction of the capture probe antibody with thiol-bound DSP linker between 1000 and 3500 cm^-1^. Figure 4A shows the peak observed at 1310 cm^-1^ of DSP before antibody incubation indicating the symmetric C-N-C (carbon-nitrogen-carbon) stretch of DSP. The peak observed at 1738 cm^-1^ in Figure 4B indicates the presence of free carboxylic acid in DSP. After antibody incubation, antibody conjugation to DSP occurs by breaking of carbon-oxygen bond within N-hydroxysuccimide (NHS) ester of DSP. Amine-reactive NHS ester reacts with primary amine of the antibody to form a stable amide bond. This occurrence is observed by the disappearance of peak at 1738 cm^-1^ due to breaking of C-O bond of NHS ester in the DSP. The appearance of 1655 cm^-1^ representing amide I band associated with C=O indicates the conjugation of cortisol and TNF-α antibody to DSP functionalized gold surface. The C-N-C stretching mode also shift from 1310 to 1316 cm^-1^ after antibody incubation suggesting that cortisol and TNF-α were successfully conjugated to the thiol linker (Figure 4A). Additionally, the broad O-H stretch at 3400 cm^-1^ confirms the Cortisol and TNF-α were successfully bound to DSP immobilized linker substrate (Figure 4C).

### Dose dependent response of sensing platform

**Figure 5.**
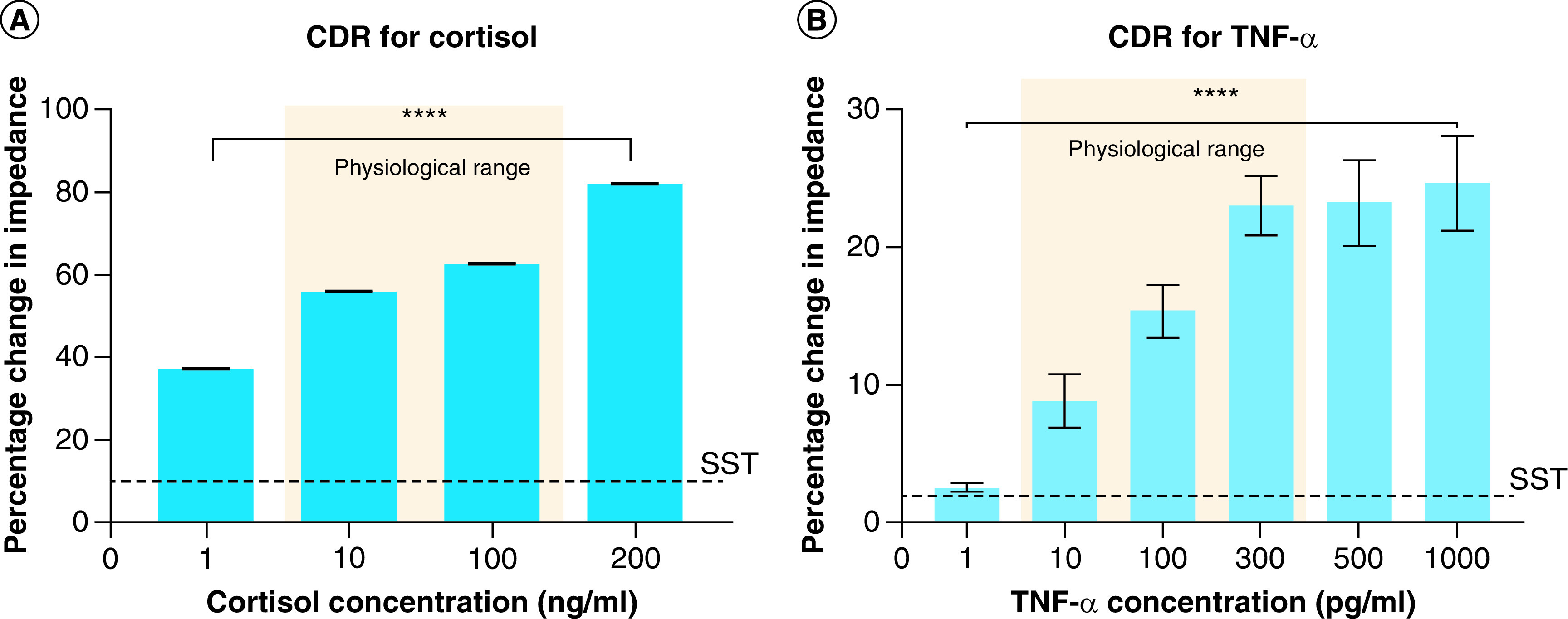
Sensor response for target biomarkers. **(A)** CDR curves for cortisol and **(B)** CDR curve for TNF-α. ****p < 0.0001. CDR: Calibrated dose response; SST: Specific signal threshold.

The performance of the affinity biosensor was characterized by building a calibration dose response curve (CDR) for varying concentrations of cortisol and TNF-α in human sweat. The CDR is represented as percentage change in impedance obtained for a given analyte concentration from a baseline concentration (without the presence of the target analyte) at 100 Hz. The analysis frequency is chosen to be 100 Hz due to maximum signal response and stable noise factor obtained within the frequency regime of interest. The calibration sensor responses of cortisol and TNF-α in human sweat are shown in Figure 5A & B, respectively. As demonstrated in Figure 5A, the percentage change in impedance varies from 37.2 ± 0.03% and 82.3 ± 0.5% for 1–200 ng/ml cortisol concentrations with a specific signal threshold (SST) of 10%. The specific signal threshold is calculated with an signal-noise ratio of 3 with the lowest detectable dose concentration 1 ng/ml lying above the SST and thus can be regarded as the LOD. We have achieved a linear dynamic range of 1–200 ng/ml which encompasses the physiological relevant range of cortisol present in human sweat and reliably distinguishes between low and elevated cortisol levels with a p-value of less than 0.001. The constructed CDR for increasing TNF-α concentrations 1–1000 pg/ml is shown in Figure 5B. The variation in percentage impedance changes is observed to be 2.5 ± 0.3% and 24.6 ± 2.4% from 1–1000 pg/ml TNF-α. The SST is computed to be 1.85% with an LOD of 1 pg/ml. The linear dynamic range is found to be 1–1000 pg/ml. The established linear dynamic range comprises of physiological TNF-α ranges found in human sweat with an ability to distinguish between low (10 pg/ml), normal (100 pg/ml) and elevated (300 pg/ml) levels of TNF-α (p < 0.001). The mechanism underlying the biosensing of cortisol and TNF-α in a complex medium such as human sweat is the charge modulations arising within the electrical double layer (EDL) formed at the electrode–sweat interface as a consequence of antibody-target analyte binding. The charge modulations induce an impedance change which can be attributed to either the charge-transfer resistance or the double layer capacitance. Here, the biosensing in capacitance dominated and the enhanced signal response obtained from the antibody-target analyte binding is due to the high charge storage capability of the double layer [36]. Typically in affinity-based assays, the double layer capacitance is enhanced due to interlinking of biomolecules of the immunoassay which causes charge modulation within the EDL [37,38]. The capacitance of the EDL increases with increasing target analyte concentrations and can thus be used to quantify sensor response.

### Long term study & temporal response of sensing platform

**Figure 6.**
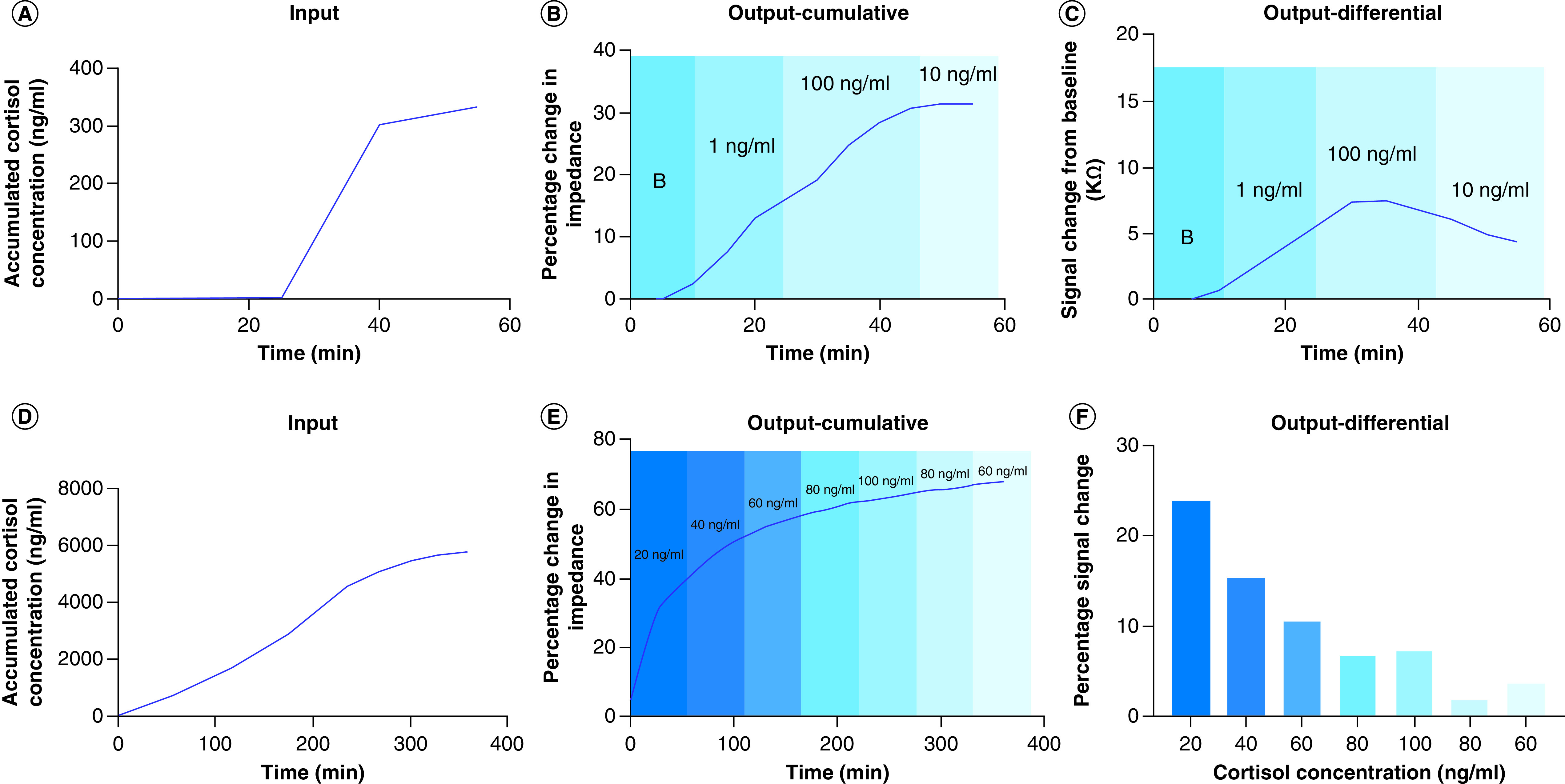
Long-term stability studies. Long-term stability study over a period of 1 h: **(A)** input for long term stability experiment versus time; **(B)** cumulative output for low, mid and high doses of cortisol; **(C)** differential output (change from baseline) highlighting rise and fall for cortisol levels. Long-term stability over a period of 6 h: **(D)** input for 6-h stability study; **(E)** cumulative output for rise and fall for cortisol levels; **(F)** differential output highlighting percentage change from baseline for cortisol concentrations over 6 h.

Continuous monitoring of biomarker levels is of paramount importance in maintaining a healthy lifestyle with an intent of providing the user with an assessment of their health status from time to time. Herein, we have demonstrated the temporal response of varying levels of cortisol on polyamide to mimic the rise and fall of cortisol as represented by the diurnal cortisol curve over a 1-h duration to assess the feasibility of continuous, dynamic monitoring of cortisol in human sweat. The cortisol biosensor was subjected to three low (1 ng/ml) and three elevated (100 ng/ml) cortisol doses spiked in human sweat to simulate the cortisol rise level; subsequently the biosensor was loaded with three intermediate (10 ng/ml) cortisol concentration to simulate the fall of cortisol level. The input cortisol dose concentration profile presented to the biosensor as accumulated dose concentrations in the 1-hour window with a sampling interval of 5 min is shown in Figure 6A. The percentage change in impedance from baseline obtained in response to the accumulated dose concentrations in human sweat, as shown in Figure 6B, indicated a change from 7.5 to 28% for the rise cycle while the percentage change observed for the fall cycle was 28–31%. In temporal accumulative dose response studies, the dynamic differential signal (DDS) change is an appropriate method to indicate the concentration being detected as affinity binding is designed for association of analytes to their receptors and sensor surface regeneration is not feasible [39]. As shown in Figure 6C, the DDS change from 0 ng/ml (baseline) to first 1 ng/ml (low dose) was observed to be 6.55 KΩ; the DDS change from last 1 ng/ml dose (low dose) to the first 100 ng/ml dose (high dose) was found to be from 5.8 to 9.7 KΩ; the DDS change from the last 100 ng/ml dose (high dose) to the first 10 ng/ml dose (intermediate dose) was found to be from 3.6 to 5.1 KΩ. The porous structure of the membrane allows for nanoconfinement of biomolecules leading to a steep rise in impedance is observed for low to high concentration dosing while the change in impedance begins to taper with high-to-intermediate dose concentration. The cortisol biosensor’s long-term temporal response to the ebb and flow of cortisol levels within 12 AM to 12 PM sleep cycle of the day is mimicked on the sensor platform as a proof-of-concept for utility as a wearable sensor. The cortisol biomarker level cycling is carried in the 6-h time period to mimic the cortisol rise and fall response with the onset of rise in cortisol at 12 PM, peaking of cortisol levels during 6 AM to 9 AM time period and fall in cortisol levels toward the late afternoon. Herein, we have chosen the cortisol levels appearing in sweat during the 6AM–12PM time period to capture the response of the sensor during the transition from rise to fall of cortisol levels. The accumulative cortisol dosing concentrations representing the rise and fall of cortisol in the 6-h sleep window of the day is shown in Figure 6D wherein dose levels increasing from 20–80 ng/ml represent the rise in cortisol, 100 ng/ml represents the peak cortisol level followed by the fall in cortisol doses from 100 to 60 ng/ml. The percentage change in impedance and the DDS change registered by the cortisol biosensor in response to the accumulative dosing input at a loading interval of 5 min are shown in Figure 6E & F. The percentage change in impedance for the 20–100 ng/ml rise cycle increases from 23 to 64%; the percentage change in impedance for the fall cycle from 100 to 60 ng/ml dose concentrations is found vary from 64 to 67%. The dynamic percentage signal change computed considering the previous dosing step as the baseline for the rise cycle decreases from 24 to 7.1%. For the fall cycle, the percentage signal change decreases from 7.1 to 2% for 100 to 80 ng/ml cortisol doses. Beyond 80 ng/ml cortisol dose, the sensor begins to show signs of saturation as the percentage signal change increases from 2 to 3.8% for 80–60 ng/ml cortisol dose. The cortisol biosensor can detect the cortisol rise and fall dose levels presented to it over a 5.5-h duration beyond which an inflection point is reached indicating slow saturation of the immobilized immunoassay. The developed biosensing platform demonstrated in this work is dynamically responsive to cortisol rise and falls levels over a period of 5.5 h continuously thus enabling early detection of circadian dysregulation and understanding its connection with the etiology and pathophysiology of disorders.

### Cross-reactivity of sensing platform

**Figure 7.**
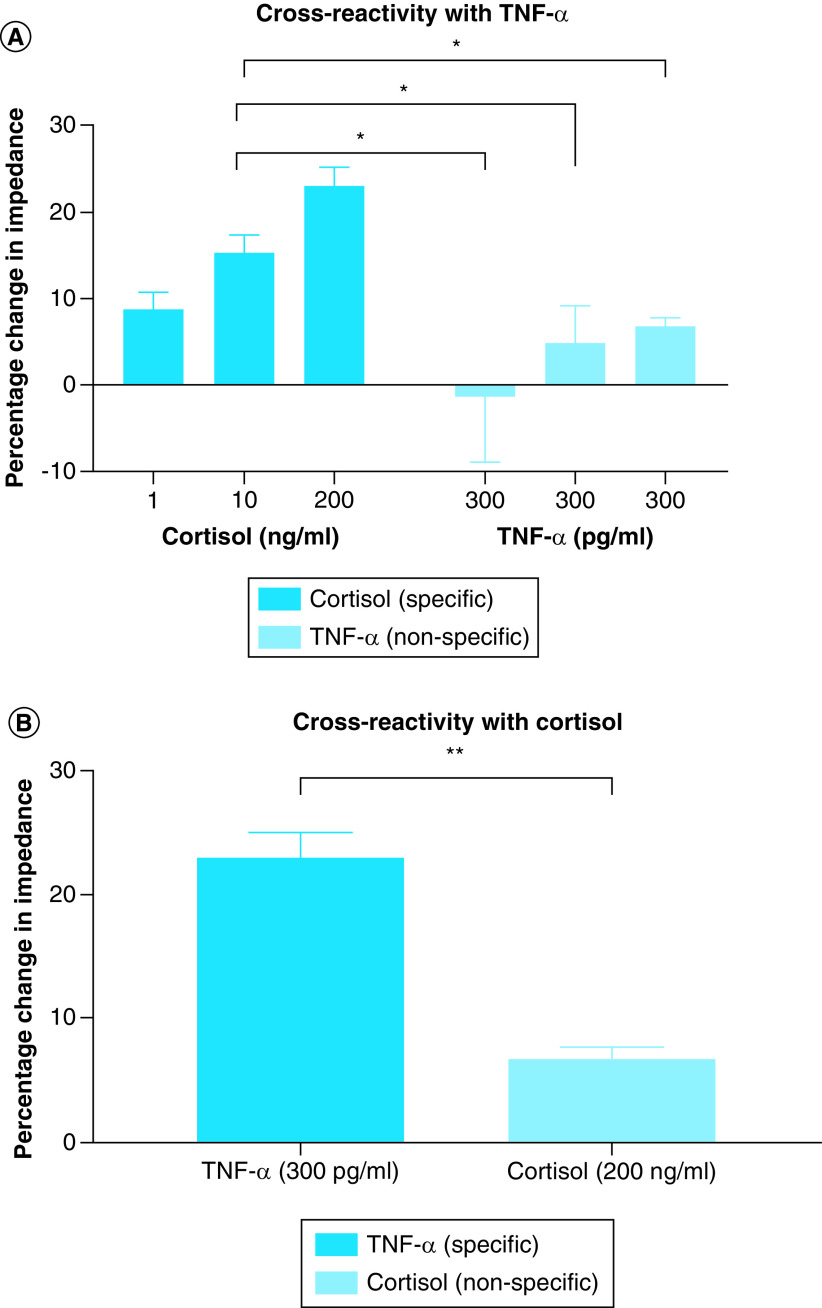
Cross-reactivity studies. **(A)** Cross-reactivity on cortisol antibody immobilized platform using TNF-α (nonspecific). **(B)** Cross-reactivity on TNF-α antibody immobilized platform using cortisol (nonspecific) molecule. *p < 0.05; **p < 0.01.

As discussed earlier, an antibody system is functionalized on the sensor surface which binds to the target molecule creating a response signal. However, there are certain instances when the antibody system might show some cross-reactivity between the signaling molecules due to inaccurate binding between the antigen epitope and antibody paratope. Sweat has a plethora of interferent molecules like hormones, proteins, urea, lactic acid, creatinine and lactic acid for example, that may be capable of generating such a false-positive response. Previous studies performed to characterize the selectivity of the system for similarly structured compounds has confirmed that the capture probes are specific for the specific molecule, for example, cortisol. In this section, as this is a multibiomarker detection platform, the cross-reactivity signal between the two molecules of interest was evaluated. This is presented in Figure 7. This study was performed in pooled human sweat, so that the biological fluid driven variance can also be accounted for while evaluating the cross-reactivity of the sensing platform. The two graphs illustrated in Figure 7 represent testing on two separate biosensors, one which is immobilized with cortisol antibody as depicted in Figure 7A and the second graph is immobilized with TNF-α antibody as shown in Figure 7B. For the sensor demonstrated in Figure 7A, three high doses (300 pg/ml*3) of TNF-α were consecutively introduced onto the functionalized sensor surface and the signal impedance response was recorded. This nonspecific signal was compared with the specific signal response of low (1 ng/ml), medium (10 ng/ml) and high dose of cortisol (200 ng/ml). The impedance response from nonspecific TNF-α doses is below the noise threshold and it is significantly lower than that of the medium and high doses of cortisol. Physiologically we would see a spike in TNF-α levels with the occurrence of inflammation; however, under normal conditions, we would not expect to see a high biomarker spike. Since the system is an irreversible binding model, the final response is a cumulative signal. From the results, it can be observed that after three subsequent high doses of TNF-α, the final cumulative signal is significantly lower than the cortisol doses with a p-value < 0.05. This indicates that the system can sensitively differentiate between the two molecules and recognize the target molecule. Similarly, the reverse analysis was carried out to evaluate the sensitivity and selectivity of TNF-α capture probe functionalized membrane surface. The cross-reactivity for TNF-α antibody immobilized surface has been illustrated in Figure 7B. This is especially important because out of the two molecules, the physiological levels are a magnitude lower for TNF-α than cortisol. Thus, it is imperative that the signal for cortisol, which will physiologically be present in sweat in higher concentration, does not cross react with the TNF-α response. We can observe that for high concentrations of both cortisol and TNF-α, the signal response is significantly higher for TNF-α (specific) molecule as compared with cortisol (nonspecific molecule). The change for TNF-α is approximately 23% from baseline (unspiked pooled human sweat) and the change for high dose of cortisol is approximately 7% from the baseline. The significance tests were carried out by performing a *t*-test analysis with confidence condition of α = 0.05. Thus, the sensing platform has specificity for the target biomarker of interest and can differentiate it from the nonspecific target.

## Conclusion

This work demonstrates the feasibility of using a sweat-based platform for tracking the intricate relationship between the endocrine and inflammatory pathway using sweat based biomarkers-cortisol and TNF-α. A detailed analysis for the optimal electrode design and platform wicking is provided using COMSOL Multiphysics simulations. Binding characterization studies performed using Fourier-transform infrared spectroscopy validated the immunochemistry used for performing affinity-based detection. Sensor showed enhanced sensitivity to biomarker concentrations in sweat with a limit of detection of 1 ng/ml for cortisol and 1 pg/ml for TNF-α. Dynamic range of the sensor encompassed the physiologically relevant ranges with low noise. Additionally, long term stability of the sensing platform was demonstrated with continuous measurements to understand the temporal response of the sensor to rise and fall cortisol concentrations in accordance with the cortisol diurnal cycle over a diagnostically relevant time period. Finally, cross-reactivity studies confirm the specificity and selectivity of the sensor for the target biomarker. In conclusion, this work uses a novel, electrochemical wearable platform to offer enhanced sensitivity and improved sensor stability to map the endocrine-inflammatory relationship toward advancement of chronic disease diagnosis and management.

## Future perspective

The focus of healthcare tools has shifted from bulky, expensive diagnostic equipment to rapid, economical and miniaturized diagnostic tools. There is a rise in patient-centric treatment approach which leads to increase in the demand of point-of-use devices. These point-of-use devices assist with performing healthcare related tasks on a miniaturized platform like a chip. In the next few years, there will be a significant change in the appearance of healthcare technologies, where lab-on-a chip devices will be taken into consideration as a predominant healthcare choice. Also, with the growing awareness in people for monitoring their health, for example, smart watches etc., self-monitoring devices will simplify the arduous task of pricking for blood to obtain biomarker levels, by employing noninvasive biofluids instead. However, there are several challenges that need to be tackled toward creating highly sensitive wearable sensing/detection platforms. These include optimizing for on-body device use, system integration using hardware, improving analytical reliability of results and improving accuracy and stability.

**Summary points**

- Sensor uses sweat based electrochemical detection to perform biomarker level quantification of cortisol and TNF-α to track the endocrine-inflammatory relationship.
- COMSOL Multiphysics^®^ simulations highlight the optimal electrode design and the wicking capabilities of the hydrophilic nanoporous membrane. This is key for sweat based detection where passive sweating is used as a mode of sample collection. The platform uses ultralow volumes of 3 μl.
- The sensing platform is flexible, hydrophilic and electrochemically stable. The limit of detection of the system is 1 ng/ml for cortisol and 1 pg/ml for TNF-α. The dynamic range of operation for the sensor is 1–200 ng/ml for cortisol and 1–1000 pg/ml for TNF-α. This shows robustness beyond the characteristic diagnostic ranges of 8–141 ng/ml for cortisol and 9–362 pg/ml for TNF-α.
- Long term stability studies highlight the ability of the sensing platform to capture the diurnal or inflammation related cycling of biomarkers over long time periods without compromised sensitivity.
- Cross-reactivity studies indicate that the platform has the ability to distinguish between the two biomarkers of interest with good sensitivity.
