# Supplementary material for: Point-of-use sweat biosensor to track the endocrine–inflammation relationship for chronic disease monitoring
Source: Future Sci OA. 2020 Oct 22;7(1):FSO628. doi: 10.2144/fsoa-2020-0097 (PMC7787138; doi:10.2144/fsoa-2020-0097)
Supplement: Supplementary file 1 [file fsoa-07-628-s1.docx]

***Point-of-use sweat biosensor to track the endocrine-inflammation relationship for chronic disease monitoring***

***
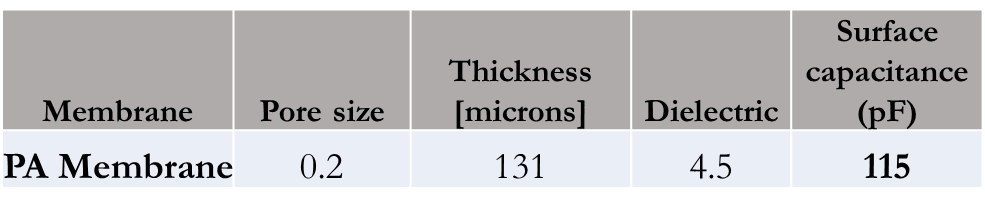
***

Table S1: Properties of Polyamide nanoporous membrane

***
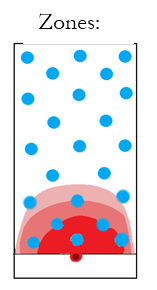
***
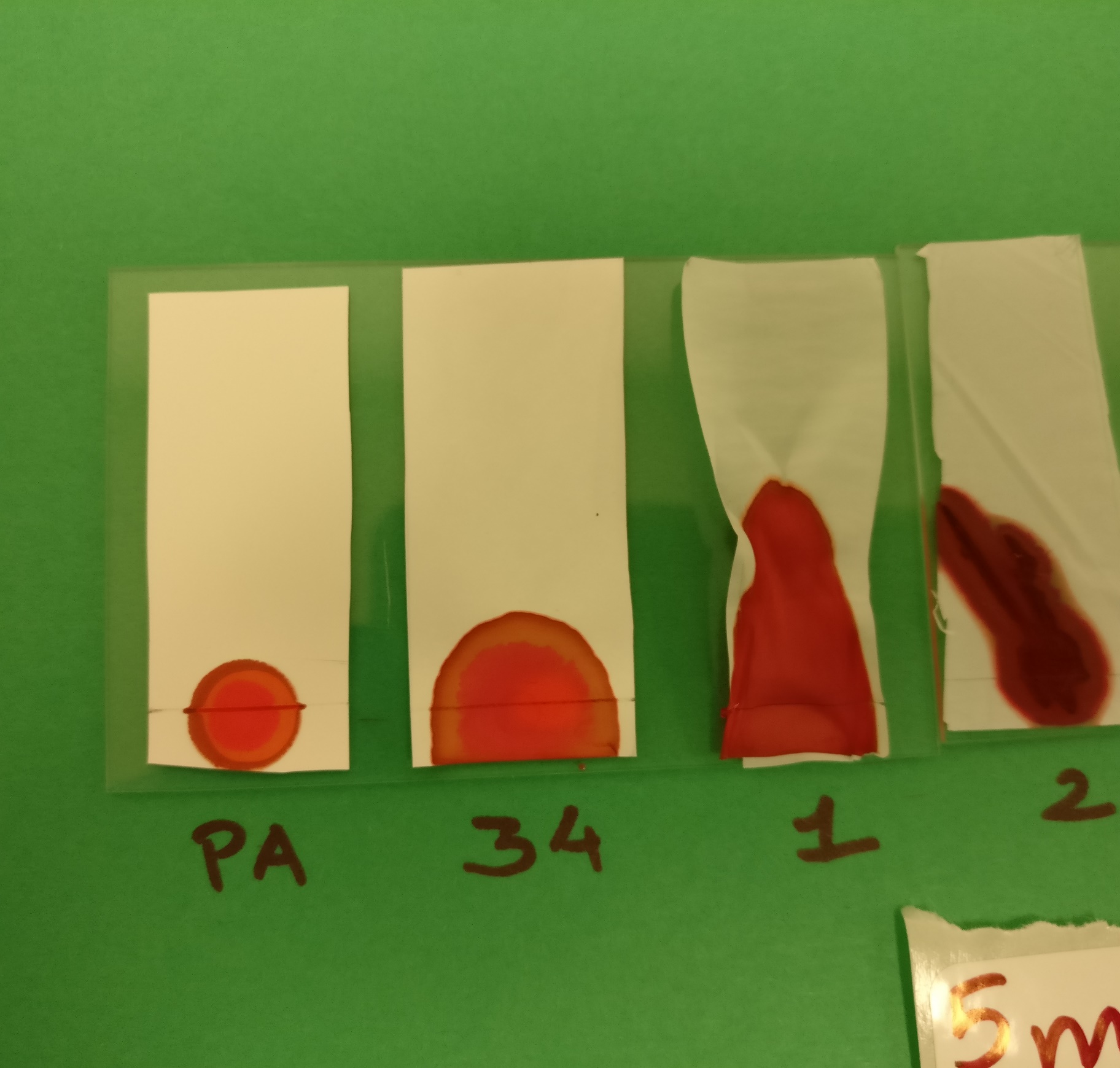


Figure S1: Zones of red dye separation on nanoporous membrane

**1. COMSOL Multiphysics simulations**

Neumann’s boundary conditions (n.J=0) were applied to the boundary while simulating the electrode distributions using PBS (Phosphate buffered saline) as an electrolyte. Equations employed for electrolyte potential and current density simulations are as follows

1. $\nabla.$J_l_ = Q_l_ , J_l_ = -σ_l_ $\nabla\phi$_l_

2.$\nabla.$J_l_ = Q_s_ , J_s_ = -σ_s_ $\nabla\phi$_s_

3.$\phi$_s_ - $\phi$_l_ = E_eq_

Js and Jl are the current density vectors (A/m2) for the electrode and electrolyte respectively. σ_s_ and σ_l_ are conductivities of electrolyte and electrode domains. Eeq depicts the potential difference during equilibrium at the electrode-electrolyte interface.


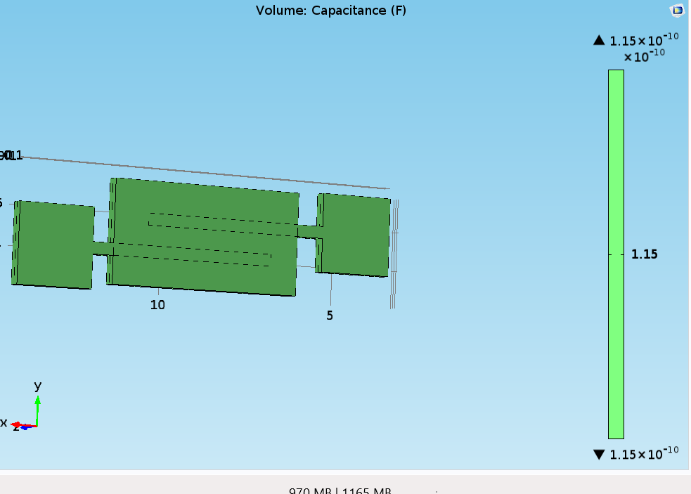


Figure S2: Distribution of capacitance based on dielectric of membrane

The equations that govern the flow related simulations are as follows:

1. Lucas Washburn equation

$$ks=\frac{l\left( t \right)^{2}*\mu r}{4\gamma\cos\left( \theta\right)t}$$

where $ks$ is membrane permeability, $l\left( t \right)$ is the distance travelled by the liquid phase w.r.t time, $\mu$ is viscosity of liquid phase, $r$ is mean pore radius, $\gamma$ is surface tension, $\theta$ is contact angle, and $t$ is time period^19,20^.

1. Darcy’s law:

$$V=-\frac{ks}{\mu}\Delta P$$

where $V$ is liquid velocity and $P$ is pore contributed pressure.

1. Mass balance equation:

$$\frac{\partial(\varepsilon_{p}c_{i})}{\partial t}+\nabla.J_{i}+u.\nabla c_{i}=R_{i}$$

$$J_{i}=-D\nabla c_{i}$$

Where, c_i_ is the concentration of the species (SI unit: mol/m^3^), D denotes the diffusion coefficient (SI unit: m^2^/s), R_i_ is a reaction rate expression for the species (SI unit: mol/(m^3^·s)), u is the velocity vector (SI unit: m/s), and $\varepsilon_{p}$ is porosity of the nanoporous membrane. This uses principles of diffusion and convection for transport.

Constant velocity profile is maintained at inlet boundaries, no flux condition is applied on the membrane boundaries. Assumptions include:

1. Uniform cross-sectional area of porous media
2. No frictional or inertial loss contributions
3. Homogeneous porous media
4. Atmospheric pressure and room temperature conditions were applied as experimental conditions.
